# Basiliximab for the therapy of acute T cell–mediated rejection in kidney transplant recipient with BK virus infection: A case report

**DOI:** 10.3389/fimmu.2022.1017872

**Published:** 2022-09-23

**Authors:** Tingting Chen, Xiaoyu Li, Jina Wang, Xuanchuan Wang, Tongyu Zhu, Ruiming Rong, Cheng Yang

**Affiliations:** ^1^ Department of Pharmacy, Zhongshan Hospital, Fudan University, Shanghai, China; ^2^ Department of Urology, Zhongshan Hospital, Fudan University, Shanghai, China; ^3^ Shanghai Key laboratory of Organ Transplantation, Shanghai, China; ^4^ Department of Transfusion, Zhongshan Hospital, Fudan University, Shanghai, China; ^5^ Zhangjiang Institute of Fudan University, Shanghai, China

**Keywords:** basiliximab, acute T cell–mediated rejection, BK virus, kidney, transplantation

## Abstract

A 66-year-old Chinese man underwent a deceased donor kidney transplantation. Induction-immunosuppressive protocol consisted of basiliximab (BAS) and methyl prednisolone (MP), followed by maintenance immunosuppression with cyclosporin (CsA), mycophenolate mofetil (MMF), and prednisone (PED). The patient’s post-transplantation course was almost uneventful, and the graft was functioning well [serum creatinine (Scr) 2.15 mg/dL]. The MMF and CsA doses were decreased 1-month post-operative as the BK virus activation was serologically positive. His Scr was elevated to 2.45 mg/dL 45 days after the transplant. A graft biopsy showed BKV nephropathy (BKVN) and acute T cell–mediated rejection (TCMR) Banff grade IIA (I2, t2, ptc2, v1, c4d1, g0, and SV40 positive). The conventional anti-rejection therapy could deteriorate his BKVN, therefore, we administered BAS to eliminate activated graft-infiltrating T cells and combined with low-dose steroid. He responded well to the therapy after two doses of BAS were given, and the kidney graft status has been stable (recent Scr 2.1 mg/dL).

## Introduction

Basiliximab (BAS), one of the agents frequently used for induction therapy in kidney transplantation, is a chimeric (human/mouse) monoclonal antibody (mAb) directed against a key component of interleukin-2 receptor (IL-2R), preventing normal T-cell proliferation and, thereby, the progression of acute cellular rejection (ACR) (1). Induction therapy using BAS significantly decreased incidence of acute rejection with minimal side effects in kidney transplantation.

Very few studies have reported the treatment of BAS in acute rejection regarding effectiveness and safety with ambiguous signal for a benefit of IL-2R mAbs. Above the most of those existing reports, BAS was used as rescue therapy in acute rejection. The efficacy of BAS in ongoing acute rejection has not been proven. Herein, we present a case of BAS for the successful therapy use for acute rejection following kidney transplantation, thereby avoiding the use of anti-thymocyte globulins (ATG), or large dose of glucocorticoids. We report this case to bring attention to this application of BAS to provide reference for safer acute rejection treatment.

## Case report

**Figure 1:**
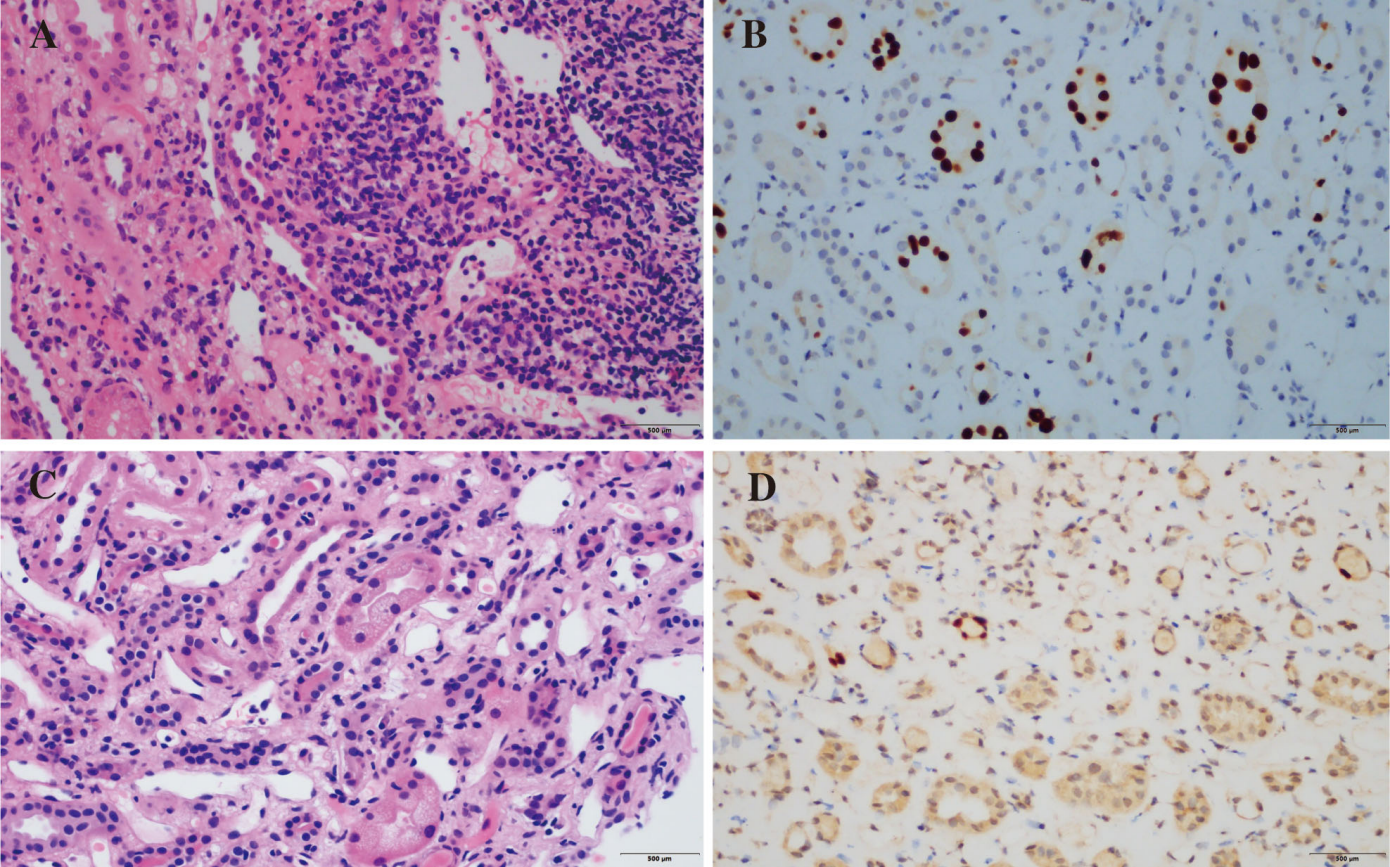
Pathological findings of renal allograft biopsy. Pathological findings before BAS treatment. Numerous graft-infiltrating cells can be seen in the renal interstitium (i2) (**A**, HE staining). Nuclear expression of SV40-T antigen (**B**, immunohistochemistry). The second renal allograft biopsy. Microvacuolar degeneration of renal tubular cells (**C**, HE staining). Persistent nuclear expression of SV40-T (**D**, immunohistochemistry).

**Figure 2:**
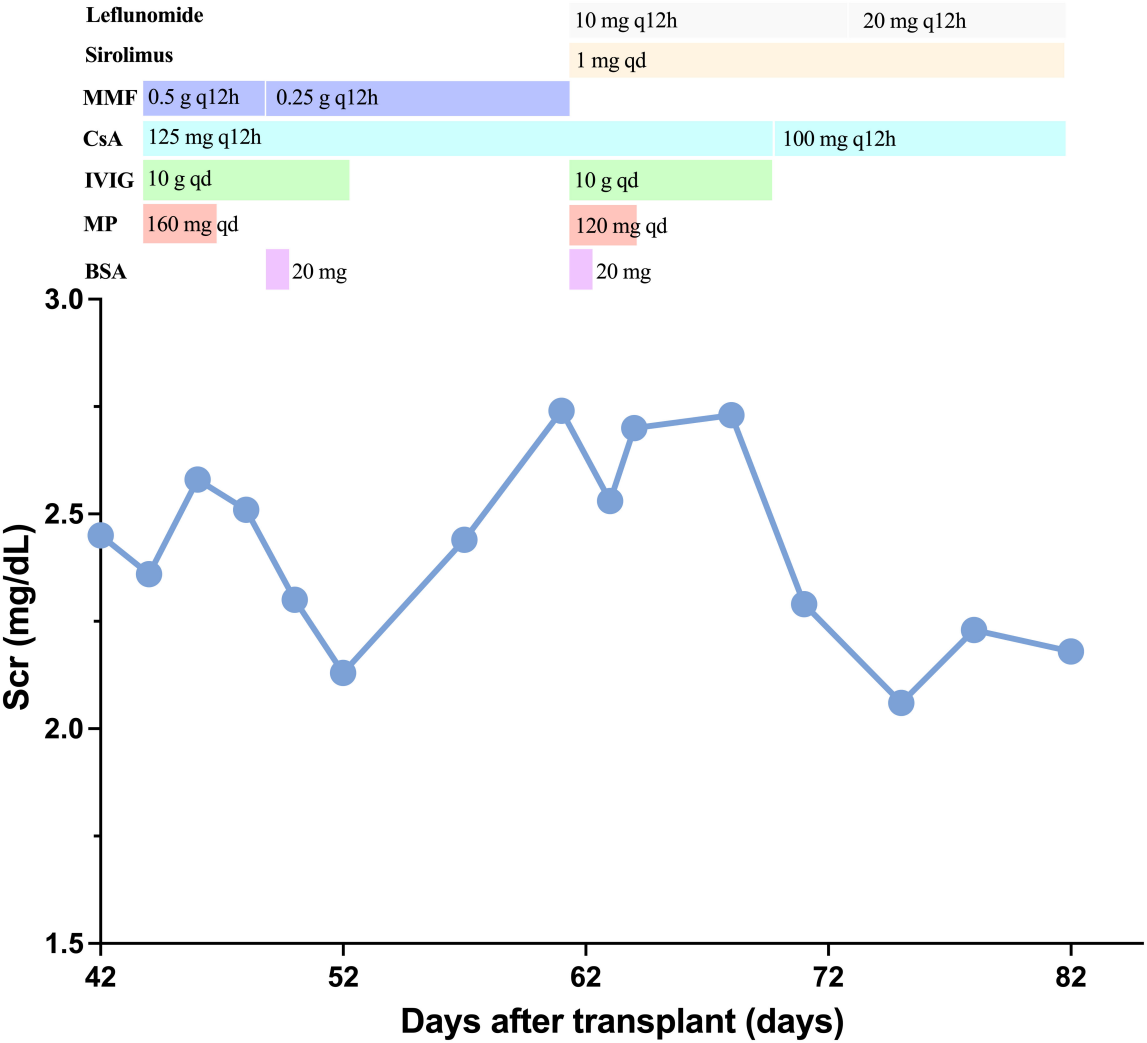
Clinical course. Change of serum creatinine and immunosuppressive agents treatment. BAS, basiliximab; MP, methyl prednisolone; IVIG, intravenous immunoglobulin; CsA, cyclosporin; MMF, mycophenolate mofetil.

**Figure 3:**
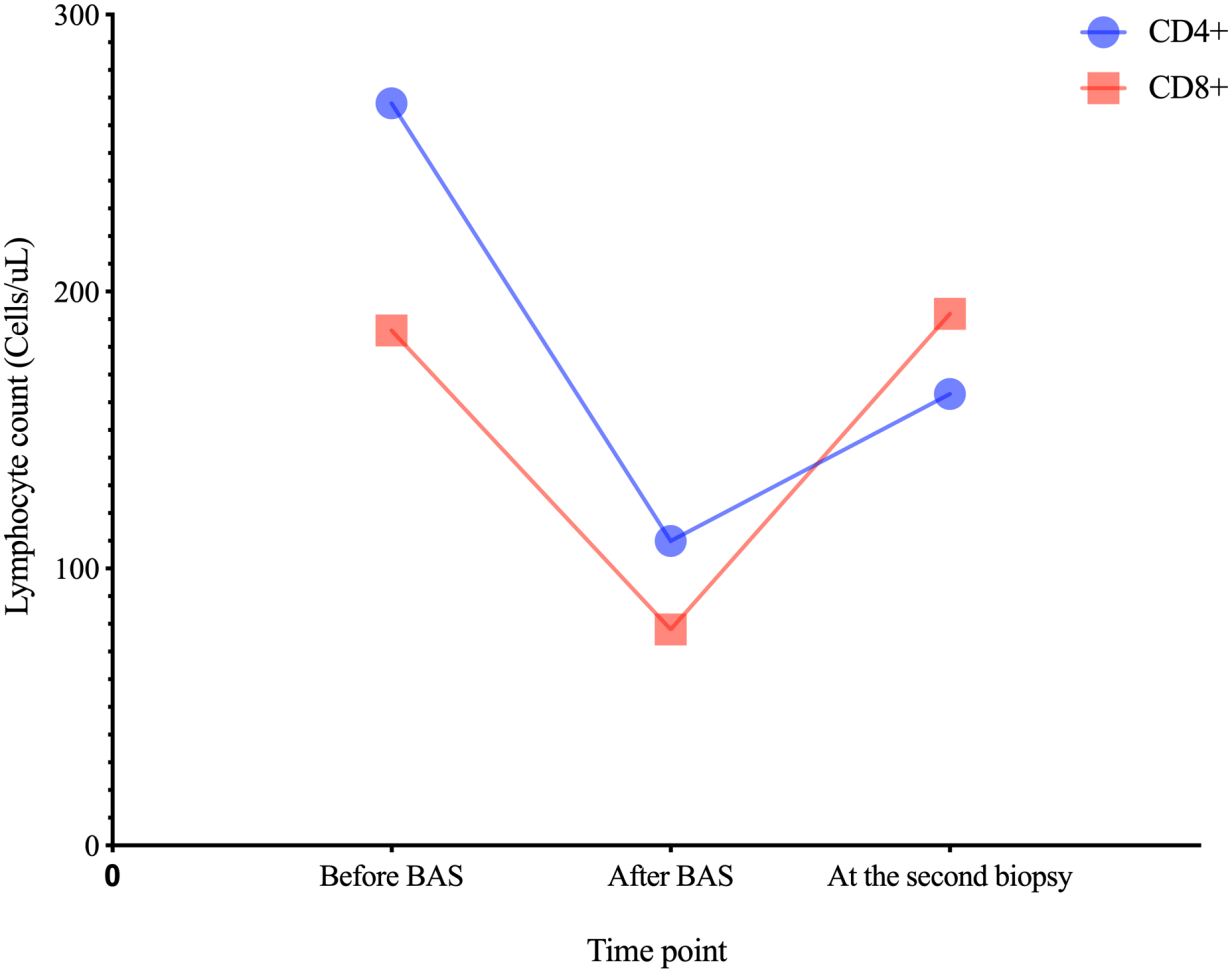
The CD4+ and CD8+ T cells levels at different time point.

A 66-year-old Chinese man, with end stage chronic kidney disease with an unknown etiology, underwent deceased donor kidney transplantation in Zhongshan Hospital, Fudan University. His other medical history included diabetes mellitus and with stable blood glucose currently. Induction-immunosuppressive protocol consisted of BAS and methyl prednisolone (MP), followed by maintenance immunosuppression with cyclosporin (CsA), mycophenolate mofetil (MMF), and prednisone (PED) on post-operative day 1. His post-operative course had been almost uneventful, and the graft had been functioning well with the serum creatinine (Scr) decreased from 731 to 128 µmol/L (8.26–2.15 mg/dL) within the next 10 days. One month later, BK virus (BKV) was activated with BKV-DNA in urine and plasma were newly detected at 3.2 × 10^7^ and 1.33 × 10^2^ copies/ml, respectively. Thereafter, the MMF and CsA doses were decreased. Two weeks later, his Scr increased to 217 µmol/L (2.45 mg/dL), with a history of feeling unwell with oliguria. Doppler ultrasonography revealed unspoiled intragraft blood flow in interlobular arteries (resistive index: 0.74). He, then, was admitted for evaluation of elevated creatinine. A graft biopsy showed BKV nephropathy (BKVN) classified with histological pattern stage B1 according to AST-IDCOP (American Society of Transplantation Infectious Diseases Community of Practice) (2) and acute T cell–mediated rejection (TCMR) Banff grade IIA (I2, t2, ptc2, v1, c4d1, g0, SV40 positive) (**Figure 1**) (3). His IL-2 receptor was increased up to 6,131 U/ml. In addition, his immune function was suppressed severely, with the lymphocyte count decreased to 510 cells/ul. Taking the high intensity of immunosuppression and the possible further deterioration of renal allograft into consideration, ATG and high-dose steroid pulse therapy have been avoided. The anti-rejection therapy targeted acute TCMR composed of a single dose of BAS (20 mg per dose), low-dose MP (160 mg per day, continuous infusion for 3 days), intravenous immunoglobulin (IVIG), and up-dosed MMF. His Scr decreased to 188 µmol/L (2.12 mg/dL) but increased up to 242 (2.74 mg/dL), 1 week thereafter. The BKV load of urine and plasma increased to 1.26 × 10^9^ and 4.70 × 10^3^ copies/ml, respectively. The second dose of BAS was administered, along with low-dose MP (120 mg per day, continuous infusion for 3 days) and IVIG. On top of that, MMF was discontinued and replaced with sirolimus and leflunomide. Finally, he responded to the anti-rejection therapy, and Scr decreased to 182 µmol/L (2.06 mg/dL) thereafter (**Figure 2**). To prevent opportunistic infection, prophylactic Sulfamethoxazole/Trimethoprim synthesis and ganciclovir were administered throughout the anti-rejection therapy, and thereafter, for 3 months. Three months later, he was suffered with candida infection and recovered through anti-bacterial therapy. On his 7 months follow-up, he has been in good health, and the kidney graft status has been stable (recent Scr 2.1 mg/dL), but his BKV still positive with BKV load in the urine and plasma were recently detected at 1.24 × 10^7^ and 1.27 × 10^3^ copies/ml, respectively. A follow-up second renal allograft biopsy 4 months later after BAS treatment showed foci of tubular atrophy and interstitial fibrosis without any evidence of tubulitis. There was no obvious inflammatory cellular infiltration compared to the first biopsy. The biopsy features were consistent with BKVN injury (histological pattern stage A) without any evidence of acute rejection (**Figure 1**). The fluorescence-activated cell sorting (FACS) was performed by the department of clinical laboratory, the data of CD4+ and CD8+ T cells before and after BAS treatment were collected (**Figure 3**). The CD4+ and CD8+ T cells levels are lower at the second biopsy compared with the first biopsy.

## Discussion

The short-term and long-term survival of kidney allograft have been significantly improved after induction therapy used (4). However, acute rejection, as a consequence of an immune response of the host to destroy the graft, is still hard to avoid and to associate with poor shorter and longer term kidney transplant outcome (5).

This patient had biopsy-proved acute rejection along with BKVN. He also had an over immunosuppression situation with the peripheral blood lymphocyte was 0.2 × 10^9^/L. ATG and large dose steroid pulse therapy seemed to be the first choice, but we had to concerned about the high risk of BKVN deterioration and other serious opportunistic infection, as the intensity of immunosuppression correlates with recurrent BKVN after transplantation (2).

BAS has been prescribed to effectively prevent acute rejection as induction therapy in kidney transplantations until now. It exerts its immunosuppressive effects through competitive antagonism of the alpha subunit of the high-affinity IL-2 receptor, effectively preventing the IL-2–mediated stimulation of lymphocytes, a critical event in the process of ACR (1). However, its role in treating pre-existing rejection has not been elucidated. Several research studied the efficiency of IL-2R on reversing an established rejection (6–17). These studies indicated that IL-2R mAb could be used safely and effectively in various situations among solid transplantation recipients, without increasing the incidence of bacterial/fungal/CMV infection, such as for steroid-resistant rejection (SRR) and rejection in HCV-positive recipients after liver transplantation, SRR, severe acute-hybrid rejection, and acute rejection after kidney transplantation. To better appreciate its role of potential therapy for ongoing acute rejection in transplant recipients, we performed a literature search (**Table 1**), which revealed only 11 previously reported studies in solid organ transplant recipients. Our patient received BAS for anti-acute TCMR therapy as an empiric decision, and its efficacy was unexpected. The possible anti-rejection effects of BAS may be by blocking the IL-2R of the antigen-activated T lymphocytes after partial suppressions of the activated immune system with steroids (15).

**Table 1:** Summarization of current literatures about IL-2R monoclonal antibody use in treatment of acute rejection after solid organ transplantation.

Published Years	Country	Type of study	Allograft	Cases number	IL-2R mAb	Indication	Dosage regimen	Outcome
2014 (7)	Japan	Retrospective case series	Liver	13	BAS	ASRR: 8 AR-HCV positive: 5	20 mg at day 1 and day 4	Reversed: 11 No reversed: 2 In patients with HCV infection, there was no increase in the viral load, except 1 described above who died
2014 (8)	Japan	Observational study	Liver	7	BAS	SRR in pediatric patients with ALF	10 mg at day 0 and day 4: 4 10 mg at day 0: 3	Reversed and alive: 6; No reversed and re-transplantation: 1
2014 (6)	China	Retrospective case series	Liver	9	BAS: 7 Daclizumab: 2	SRAR	BAS group: 40 mg with an interval of 4 days + MMF 0.75g bid: 7 Daclizumab group: 2	Effectively reversed: 3; Pathology recovery: 1; Not recovery: 5
2012 (9)	Spain	Case report	Liver	1	BAS	ASRR	BAS 40 mg with an interval of 4 days	Reversed
2009 (11)	Japan	Case report	Kidney	1	BAS	Acute TCMR	A single dose (20 mg)	Reversed
2008 (12)	Japan	Case report	Kidney	1	BAS	Acute-hybrid rejection	A single dose (20 mg)	Reversed
2005 (13)	UK	Retrospective case series	Liver	25	Daclizumab: 19 BAS: 6	SRR	BAS group: 20 mg/dose, with a repeat infusion 3 to 5 days later Daclizumab group: 1 mg/kg, with repeat infusions 7 to 14 days later	For patients with ACR (n=16): resolution: 12 (75%), progressive hepatic dysfunction developed: 4 (25%); For patients with established chronic rejection (n=9): persistent chronic graft dysfunction: 4, requiring repeat transplantation: 3, died: 2
2003 (14)	Singapore	Prospective study	Liver	7	BAS	SRR in pediatric recipients	Two doses of BAS (10 mg, 3-7 days apart): 5 Single dose: 2	Effectively reversed: 3 Pathology recovery: 2 Not recovery: 2
2001 (15)	Singapore	Case report	Kidney	1	BAS	SRAR	20 mg/dose, with a repeat infusion 4 days later	Effectively reversed
2001 (16)	USA	Case series	Kidney: 12 Kidney/ pancreas: 2 Liver: 4	18	BAS	AR	40 mg were infused twice over 30 min on day 1 and 4 to 6 days later	Successfully reversed: 13 (72.2%)
1990 (17)	France	Randomized controlled trial	Kidney	10	33B3.1	AR (ACR: 9)	20 mg/d × 2d, followed by 10 mg/d for additional days. In case of mAb ineffectiveness at day 5, mAb was discontinued and a rescue treatment of corticosteroid boluses was given.	Immediately respond: 2 Stabilization of Scr only: 4 Remaining increase of Scr levels at day 5: 4.

BAS, basiliximab; ASRR, acute steroid-resistance rejection; AR, acute rejection; HCV, hepatitis C virus; SRR, steroid-resistance rejection; ALF, acute liver failure; SRAR, steroid-resistance acute rejection; MMF, mycophenolate mofetil; TCMR, T cell–mediated rejection; ACR, acute cellular rejection; Scr, serum creatinine.

Three points are well worth considering in our presented case (1). A single dose of BAS may be not enough for anti-acute TCMR therapy. Although two cases reported successful recovery of renal transplant rejection with a single dose of BAS, these two patients were also treated with Muromonab CD3 (OKT3) (11, 12). This case in our study shows a fast recovery of serum creatinine after a single dose of BAS administration. But the Scr increased again a week later and decrease after the second dose was administrated (2). High IL-2R may be one of the possible reasons that contributed to the success of the treatment in this patient. A significant increase with the expression of IL-2R could be observed in the transplantation recipients with AR. The IL-2R of the patient in our case was very high, reaching to over 6,000 U/ml. It needs to be further explored whether the high expression of IL-2R is associated with the prognosis of BAS therapy (3). Though BAS appears safe when used with standard immunosuppression at induction, caution is required when it is used in patients who are at risk for overimmunosuppression to prevent graft loss from rejection.

## Conclusion

In conclusion, we have shown a case of TCMR in kidney transplantation recipient with BKVN, which implies the efficiency of BAS in anti-TCMR treatment. The patient avoided subsequent graft loss after anti-CD25 mAb therapy without any progression of BKVN.

## Data availability statement

The raw data supporting the conclusions of this article will be made available by the authors, without undue reservation.

## Ethics statement

The studies involving human participants were reviewed and approved by Ethics Committee of Zhongshan Hospital, Fudan University. The patients/participants provided their written informed consent to participate in this study. Written informed consent was obtained from the individual(s) for the publication of any potentially identifiable images or data included in this article.

## Author contributions

Conceptualization, TC and CY; Investigation, XL, JW, and XW; Resources, RR; Data Curation, JW and XW; Writing – Original Draft Preparation, TC and XL; Writing – Review & Editing, CY and RR; Project Administration, CY. All authors contributed to the article and approved the submitted version.

## Funding

This study was supported by the National Natural Science Foundation of China (82170765 to CY, 81970646 to RR), the 2019 Shanghai Youth Talent Development Program (to CY).

## Conflict of interest

The authors declare that the research was conducted in the absence of any commercial or financial relationships that could be construed as a potential conflict of interest.

## Publisher’s note

All claims expressed in this article are solely those of the authors and do not necessarily represent those of their affiliated organizations, or those of the publisher, the editors and the reviewers. Any product that may be evaluated in this article, or claim that may be made by its manufacturer, is not guaranteed or endorsed by the publisher.
